# Temporal proteome dynamics of *Clostridium cellulovorans* cultured with major plant cell wall polysaccharides

**DOI:** 10.1186/s12866-019-1480-0

**Published:** 2019-06-03

**Authors:** Shunsuke Aburaya, Wataru Aoki, Kouichi Kuroda, Hiroshi Minakuchi, Mitsuyoshi Ueda

**Affiliations:** 1 Division of Applied Life Sciences, Graduate School of Agriculture, Kyoto University, Sakyo-ku, Kyoto, Japan; 2 Research Fellow of the Japan Society for the Promotion of Science, Sakyo-ku, Kyoto, Japan; 3 Kyoto Integrated Science and Technology Bio-Analysis Center, Shimogyo-ku, Kyoto, Japan; 4 JST-PRESTO, Chiyoda-ku, Tokyo, Japan; 5 Kyoto-monotech, 1095, Shuzei-cho, Kamigyo-ku, Kyoto-shi, Kyoto, 602-8155 Japan

**Keywords:** *Clostridium cellulovorans*, Proteome analysis, Temporal analysis, Quantitative analysis

## Abstract

**Background:**

*Clostridium cellulovorans* is a mesophilic, cellulosome-producing bacterium containing 57 genomic cellulosomal enzyme-encoding genes. In addition to cellulosomal proteins, *C. cellulovorans* also secretes non-cellulosomal proteins to degrade plant cell wall polysaccharides. Unlike other cellulosome-producing *Clostridium* species, *C. cellulovorans* can metabolize all major plant cell wall polysaccharides (cellulose, hemicelluloses, and pectins). In this study, we performed a temporal proteome analysis of *C. cellulovorans* to reveal strategies underlying plant cell wall polysaccharide degradation.

**Results:**

We cultured *C. cellulovorans* with five different carbon sources (glucose, cellulose, xylan, galactomannan, and pectin) and performed proteome analysis on cellular and secreted proteins. In total, we identified 1895 cellular proteins and 875 secreted proteins. The identified unique carbohydrate-degrading enzymes corresponding to each carbon source were annotated to have specific activity against each carbon source. However, we identified pectate lyase as a unique enzyme in *C. cellulovorans* cultivated on xylan, which was not previously associated with xylan degradation. We performed *k*-means clustering analysis for elucidation of temporal changes of the cellular and secreted proteins in each carbon sources. We found that cellular proteins in most of the *k*-means clusters are involved in carbohydrate metabolism, amino acid metabolism, translation, or membrane transport. When xylan and pectin were used as the carbon sources, the most increasing *k*-means cluster contained proteins involved in the metabolism of cofactors and vitamins. In case of secreted proteins of *C. cellulovorans* cultured either on cellulose or xylan, galactomannan, and pectin, the clusters with the most increasing trend contained either 25 cellulosomal proteins and five non-cellulosomal proteins or 8–19 cellulosomal proteins and 9–16 non-cellulosomal proteins, respectively. These differences might reflect mechanisms for degrading cellulose of other carbon source. Co-abundance analysis of the secreted proteins revealed that proteases and protease inhibitors accumulated coordinately. This observation implies that the secreted protease inhibitors and proteases protect carbohydrate-degrading enzymes from an attack from the plant.

**Conclusion:**

In this study, we clarified, for the first time, the temporal proteome dynamics of cellular and secreted proteins in *C. cellulovorans*. This data will be valuable in understanding strategies employed by *C. cellulovorans* for degrading major plant cell wall polysaccharides.

**Electronic supplementary material:**

The online version of this article (10.1186/s12866-019-1480-0) contains supplementary material, which is available to authorized users.

## Background

Human lifestyle relies vastly on fossil fuels, and excessive consumption produces high CO_2_ emissions resulting in environmental pollution [1–4]. Thus, the demand for alternative and sustainable energy sources is increasing [1–4]. Significant attention has been paid to second-generation biofuels derived from inedible biomass [1–4]. The saccharification of polysaccharides derived from plant cell walls is the limiting step in the production of second-generation biofuels from inedible biomass [5]. Plant cell walls are mainly composed of three polysaccharides: cellulose, hemicelluloses (xylan, xyloglucan, glucuronoxylan, glucomannan, and galactomannan), and pectins [6, 7]. It is necessary to degrade all types of plant cell wall-derived polysaccharides for efficient biofuel production.

Cellulose, a major component of plant cell walls, is the most abundant polysaccharide in lignocellulosic biomass. It is a linear, unbranched homopolymer composed of ß-1,4-glycosidic bonds [8]. Xylan, a major component of hemicellulose in hard wood, straw, and grass, is a branched heteropolymer consisting of xylose and arabinose. Some xylan residues possess an acetylated or methylated glucose moiety. Galactomannan, a major component of hemicellulose in soft wood, is a branched monopolymer consisting of mannose. Mannose residues are linked to galactose in galactomannan, and the degree in the substitution of galactose differs in the plants. Pectins are the most complex family of polysaccharides in the plant cell wall. They are composed of homogalacturonan, rhamnogalacturonan I, and rhamnogalacturonan II [7].

In this study, we focused on the anaerobic bacterium *Clostridium cellulovorans*, which was originally isolated from wood fermenters in 1984 [9]. *C. cellulovorans* can degrade all types of major plant cell wall polysaccharides (cellulose, hemicelluloses, and pectins) using the cellulosome [10]. Cellulosome is a multienzyme complex composed of scaffoldins and enzymes [11, 12]. Scaffoldins are involved in the assembly of other cellulosomal proteins and have cohesin domains that interact with a dockerin domain [11, 12]. Cellulosomal proteins contain the dockerin domain and an enzymatic domain [11, 12]. Consecutive cohesin domains are proximally positioned in the cellulosomal proteins. The synergistic reaction of the assembled carbohydrate-active enzymes in the cellulosome allows for a higher activity in the degradation of polysaccharides compared with free carbohydrate-active enzymes [11, 12].

*C. cellulovorans* has 57 genomic cellulosomal genes with dockerin domain-coding sequences, which comprise of 25 genes encoding the glycoside hydrolase (GH) family of proteins, two carbohydrate esterase (CE) family proteins, and four polysaccharide lyase (PL) family proteins [13–15]. In addition to cellulosomal proteins, *C. cellulovorans* secretes several carbohydrate-active enzymes without a dockerin domain (non-cellulosomal proteins) [14, 16]. *C. cellulovorans* has 168 non-cellulosomal proteins with N-terminal signal peptides, which comprise of 89 GH, 19 CE, 9 PL, and 38 glycosyltransferase (GT) family proteins [16]. *C. cellulovorans* can degrade more types of plant cell wall polysaccharides than other *Clostridium* species because *C. cellulovorans* encodes more types of enzymes within its genome [13–15]. This wider substrate spectrum of *C. cellulovorans* [9] is a promising feature for its use in the efficient production of biofuels.

Analyzing the temporal proteome dynamics of *C. cellulovorans* upon culture using varied carbon sources will prove beneficial for a further understanding of polysaccharide degradation strategies and, consequently, improve production of biofuels by engineering metabolic pathways depending on carbon sources. A previous study on *C. cellulovorans* used a defined time point for proteomic analyses of the secreted and cellular proteins to understand mechanisms underlying polysaccharide degradation and metabolism [16, 17]. Another proteome analysis, also performed at a defined time point, analyzed signal transition and metabolism-related proteins [18]. However, these analyses could not reveal temporal dynamics of secretory proteins.

In the present study, we cultured *C. cellulovorans* on five carbon sources (cellulose, xylan, galactomannan, pectin, and glucose) and performed quantitative proteome analysis at five different time points using tandem mass tag (TMT) labeling [19]. The temporal dynamics of cellular and secreted proteins of *C. cellulovorans* allowed the identification of protein expression profiles crucial for the degradation of polysaccharides.

## Results

### Growth curve analysis

**Fig. 1:**
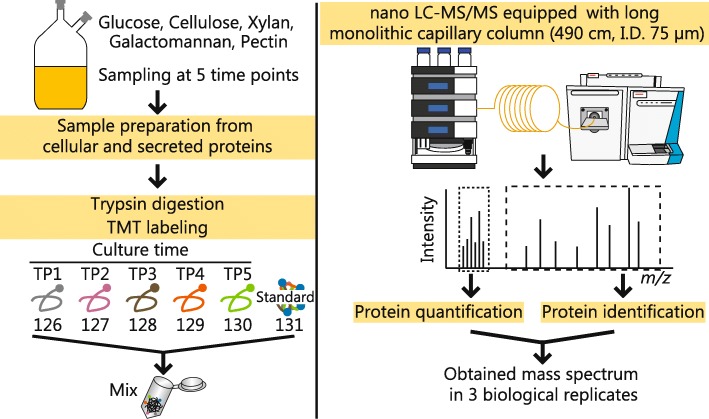
Experimental procedure of temporal quantitative proteome analysis. *C. cellulovorans* was cultured with glucose, cellulose, xylan, galactomannan, or pectin. Proteins were prepared from cell lysates at various time points. Tryptic fragments were labeled with tandem mass tag (TMT). The labeled peptides were mixed and analyzed with the nano-liquid chromatography (LC)–mass spectrometry (MS/MS) system, and the obtained mass spectra were used for protein identification and quantification. TP indicates time points at which the proteome samples were collected

**Fig. 2:**
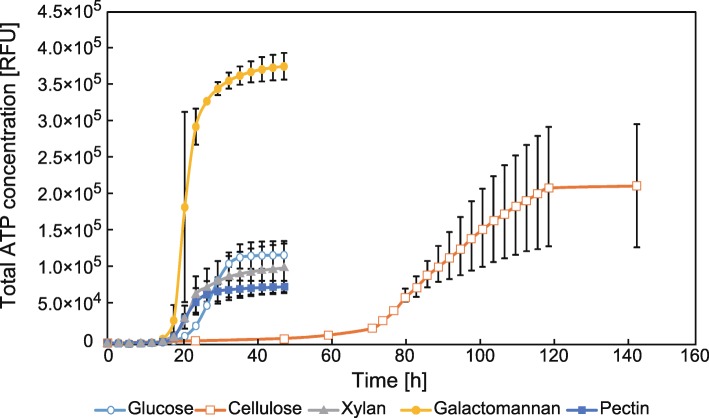
Confirmation of growth curves of *C. cellulovorans* cultured with five different carbon sources. The growth curves of *C. cellulovorans* were determined by measuring ATP concentration with five different carbon sources, glucose (open circle), cellulose (open square), xylan (closed triangle), galactomannan (closed circle), and pectin (closed square). Error bars indicate mean ± standard deviations (*n* = 3)

The experimental workflow is described in Fig. 1. First, we measured the growth curves of *C. cellulovorans* cultured using each carbon source (Fig. 2) as performed previously [20, 21]. The degradation of cellulose and xylan was confirmed in a previous report [19]. Here, we choose xylan and galactomannan as hemicelluloses because xylan is the main component of hemicellulose in hardwood [8] and galactomannan is the main component of hemicellulose in plant seeds [22]. *C. cellulovorans* was grown in all carbon sources, and it degraded, metabolized galactomannan, and produced ATP more efficiently by using galactomannan compared with other carbon sources. *C. cellulovorans* degraded cellulose very slowly. Next, we determined five sampling points as follows: lag phase (time point 1 [TP1]), early log phase (TP2), middle log phase (TP3), late log phase (TP4), and stationary phase (TP5) (Table 1). At each TP, we collected liquid culture and harvested cells for proteome analysis.

**Table 1:** Culture time point in each growth phase

	Time point 1	Time point 2	Time point 3	Time point 4	Time point 5
Growth phase	Lag	Early log	Middle log	Late log	Stationary
Glucose	13.5 h	21 h	24 h	30 h	39 h
Cellulose	24 h	60 h	75 h	81 h	144 h
Xylan	12 h	15 h	21 h	24 h	36 h
Galactomannan	12 h	18 h	19.5 h	21 h	27 h
Pectin	12 h	16.5 h	18 h	21 h	30 h

### Proteome analysis

**Fig. 3:**
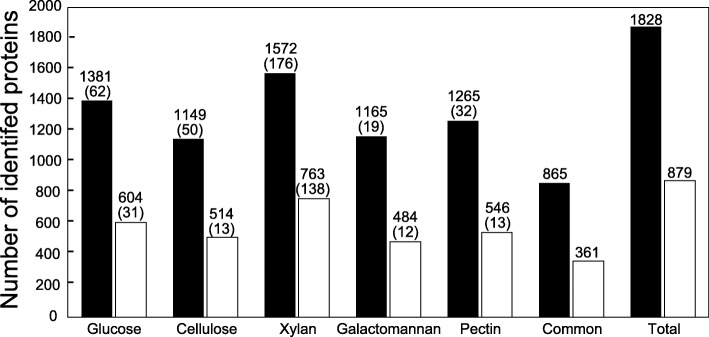
Number of identified cellular and secreted proteins (cellular, closed bar; secreted, open bar). “Common” indicates proteins identified in all carbon sources, and “Total” indicates proteins identified in any carbon source. Numbers shown in the parentheses indicate the number of specific proteins

Cellular protein samples (obtained by disrupting cell pellet) and secreted proteins (in culture supernatant obtained after centrifugation) were prepared using three biological replicates grown on each carbon source. We performed quantitative proteome analysis using TMT labeling and capillary monolith nano-liquid chromatography (LC)–mass spectrometry (MS/MS). We identified a total of 1895 cellular proteins, among which 865 were common to all carbon sources. Overall, 879 secreted proteins were identified, of which 361 were common to all carbon sources (Fig. 3). The proteome analysis covered approximately 50% of all gene products of *C. cellulovorans*; this proteome coverage is so far the highest reported in *C. cellulovorans* research [16–18]. The identified proteins and their quantitative values corresponding to carbon sources are summarized in Additional file 1. We confirmed the reproducibility of proteome analysis with coefficients of correlation. The value of coefficients of correlation is more than 0.65 in about 80% of biological replicates under all carbon sources. Values between each biological replicate are shown in Additional file 2.

### Unique identified proteins corresponding to each carbon source

We assumed that the unique identified proteins corresponding to each carbon source (Table 2) are important for the assimilation of the substrates. Particularly, we observed uniquely identified carbohydrate-degrading enzymes and cellulosomal proteins among the cellular and/or secreted proteins.

**Table 2:** Number of uniquely identified proteins corresponding to each carbon source

	Number of uniquely identified proteins in each carbon source	
	Cellular proteins	Secreted proteins
Glucose	62	31
Cellulose	50	13
Xylan	176	138
Galactomannan	19	12
Pectin	32	13

Enzymes possibly related to carbohydrate degradation among the uniquely identified proteins are shown in Table 3.

**Table 3:**
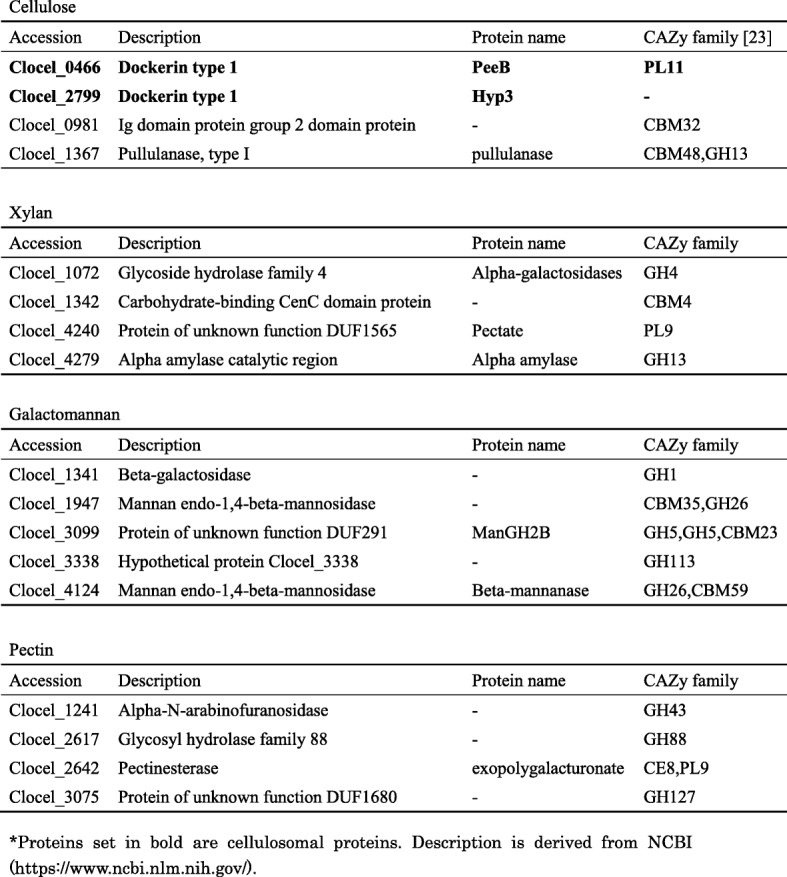
Enzymes possibly related to carbohydrate degradation among the uniquely identified proteins

Proteins set in bold are cellulosomal proteins. Description is derived from NCBI (https://www.ncbi.nlm.nih.gov/)

### *k*-means clustering analysis

**Fig. 4:**
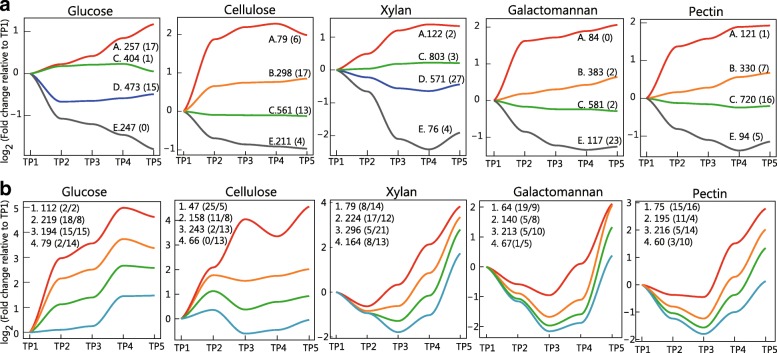
Temporal proteome changes in cellular and secreted proteins. **a** The cellular protein profiles were grouped by using *k*-means clustering. We grouped the cellular proteins in four clusters, and the protein clusters were divided into five classes as follows: cluster A, red; cluster B, orange; cluster C, green; cluster D, blue; and cluster E, gray. The number shown on cluster bars indicates the protein belonging to its cluster, and the number shown in parentheses indicates the number of identified cellulosomal proteins in each cluster. **b** The secreted protein profiles were grouped by *k*-means clustering. We grouped the secreted proteins into four clusters (1st [red], 2nd [orange], 3rd [green], and 4th [blue] clusters). The numbers on the graph indicate the number of proteins belonging to each cluster, and the number shown in parentheses indicates the number of identified cellulosomal (before slash) and non-cellulosomal (after slash) proteins

To elucidate temporal proteome dynamics for each carbon source, we performed a non-hierarchical *k*-means clustering analysis. In this analysis, we grouped the cellular and secreted proteins for each carbon source into four clusters. These clusters for each carbon source showed five trends for cellular proteins: most increasing (cluster A), slightly increasing (cluster B), stable (cluster C), slightly decreasing (cluster D), and decreasing (cluster E) (Fig. 4a). In case of secreted proteins, almost all protein clusters showed an increasing trend; however, a few protein clusters showed a decreasing trend in case of cellulose, pectin, and galactomannan (Fig. 4b).

We performed Kyoto encyclopedia of genes and genomes (KEGG) orthology analysis to categorize the functional classes of each protein cluster in cellular proteins [24, 25]. In almost all clusters of each carbon source, the top 3-ranked protein category classes were classified into carbohydrate metabolism, amino acid metabolism, translation, or membrane transport proteins (Additional file 3). In contrast, in the cluster A of pectin and xylan, proteins for the metabolism of cofactors and vitamins were ranked as top 1. Those in cluster E of xylan, proteins for energy metabolism were ranked as top 1 (Additional file 3).

### Pathway analysis of cellular proteins

We performed pathway analysis to confirm the response of *C. cellulovorans* to the different carbon sources. To this end, we plotted the fold changes in glucose at TP1 for each carbon source (Additional files 4 and 9).

Enzymes involved in the metabolism of xylan (xylan 1,4-beta-xylosidase, xylose isomerase, xylulokinase) were increased when xylan was used as a carbon source compared with glucose. Compared with growth on glucose, enzymes involved in the metabolism of galactomannan were increased when galactomannan was used as a carbon source. Compared with growth on glucose, enzymes involved in the metabolism of pectin were increased when bacteria were cultured on pectin. In summary, metabolic enzymes were increased corresponding to the available carbon source (Additional files 7 and 9).

### Co-abundance analysis of secreted proteins

**Fig. 5:**
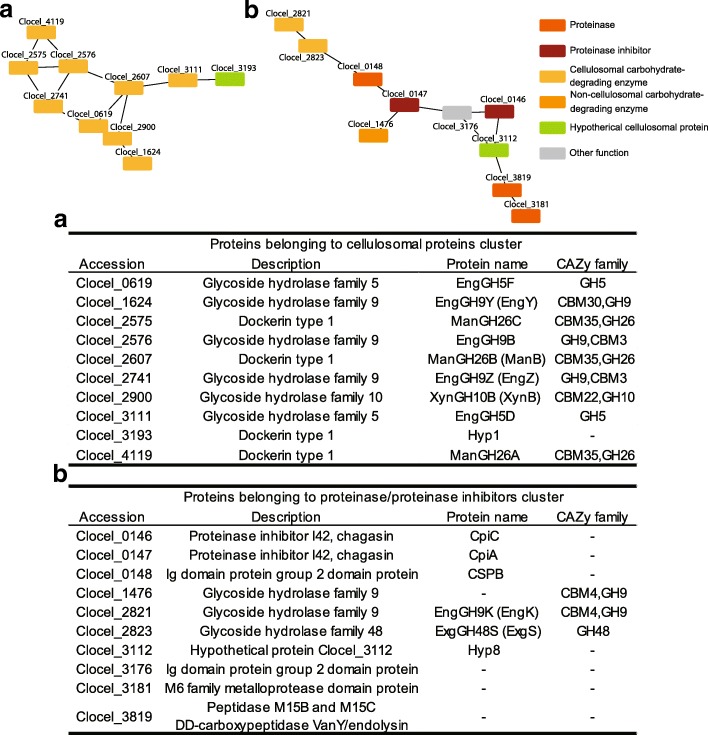
Co-abundance analysis of the secreted proteins. Two medium-sized clusters and proteins belonging to each cluster. Nodes and edges indicate proteins and their correlations. Edges indicate correlation coefficients larger than 0.80 or smaller than − 0.80. **a** Medium-sized cluster comprised cellulosomal carbohydrate-degrading enzymes. **b** Medium-sized cluster comprised proteinases, proteinase inhibitors, and some cellulosomal proteins. Description is derived from NCBI

We performed a co-abundance analysis [26] against the commonly identified secreted proteins to determine potential co-abundance patterns at all time points in an unbiased manner. Proteins that exhibit associated functions are often simultaneously accumulated [27]. Therefore, proteins with high correlation coefficients imply a functional or physical link [27]. We calculated Pearson’s correlation coefficient using R, and the potential protein co-abundance networks were visualized using Cytoscape 3.5.0 [28]. One big cluster, two medium-sized clusters, and dozens of mini clusters were observed. The big cluster included proteins related to glycolysis, amino acid synthesis, and chaperone functions. We focused on two medium-sized clusters, which included proteins with similar functions (Fig. 5). These clusters were enriched in cellulosomal proteins (cluster (a)) or proteinases/proteinase inhibitors (cluster (b)), and the abundance of proteins in these two clusters was coordinately regulated in the secreted proteins. Cellulosomal proteins in cluster (a) showed an increasing trend from *k*-means clustering analysis (Fig. 5).

In cluster (b), we identified three proteinases, two cellulosomal proteinase inhibitors, two cellulosomal carbohydrate enzymes, one non-cellulosomal carbohydrate enzyme, and one cellulosomal hypothetical protein. All proteinase inhibitors identified in the secreted proteins belonged to this protein cluster. In the cellular proteins, the correlation coefficients of proteinases and proteinase inhibitors were between 0.000 and 0.702, whereas those in the secreted proteins were higher than 0.80.

## Discussion

We performed a temporal proteome analysis after culturing *C. cellulovorans* using five different carbon sources to investigate the temporal pattern of cellular and secreted proteome profiles. We identified a total of 1895 and 879 cellular and secreted proteins, respectively.

### Unique identified carbohydrate-degrading enzymes corresponding to each carbon source

We identified two cellulosomal proteins, PeeB and Hyp3, and two non-cellulosomal proteins in the samples corresponding to cellulose as the carbon source. The hypothetical cellulosomal protein, Hyp3, was increased by about 14 times in secreted proteins (Additional file 1). The activities of these proteins are unknown, and further research is required.

We identified four unique non-cellulosomal proteins in the xylan samples. None of them have so far been annotated as xylan degradation-related proteins, and these proteins belong to the GH4, GH13, PL9 families and hypothetical proteins. Among them, Clocel_4279 showed an increasing trend in cellular proteins compared with TP1, whereas the abundance of Clocel_1342 and Clocel_4240 of TP5 increased about 10 times in secreted proteins compared with TP1. Among these, one protein (belonging to the PL9 families) is annotated as pectin-degrading enzyme in the carbohydrate-active enzymes (CAZy) database [23]. Furthermore, proteins belonging to GH4 family have alpha 1–4 galacturonase or alpha-glucuronidase activity [23, 29]. The main component of xylan from hardwood is *O*-acetyl-4-*O*-methylglucuronoxylan [30], and the side chain cannot be digested with xylanase and xylosidase. Therefore, pectin-degrading enzymes are required to degrade the side chains of *O*-acetyl-4-*O*-methylglucuronoxylan. To our knowledge, it has not been reported that the PL9 and GH4 family proteins are required for the degradation of xylan or *O*-acetyl-4-*O*-methylglucuroxylan [23, 31]. This implies that *C. cellulovorans* changes the protein profile of carbohydrate-degrading proteins, depending not only on the main chain but also on the side chain of the polysaccharides.

In samples corresponding to pectin and galactomannan carbon sources, we identified four and five non-cellulosomal proteins, respectively. These proteins are annotated as pectin- or galactomannan-degrading proteins in the CAZy database, respectively [23]. In galactomannan, Clocel_1341 showed increasing trend in cellular proteins, and the abundance of Clocel_3099 in TP5 increased about four times compared with TP1 (Additional file 1). In pectin, Clocel_2617, Cloce_2642, and Cloce_3075 showed increasing trend in cellular proteins, whereas the abundance of Clocel_2642 in TP5 increased about seven times in secreted proteins (Additional file 1).

### *k*-means clustering analysis

The *k*-means clustering of cellular proteins suggested that the abundance of cellulosomal proteins on the cell surface might not increase during the degradation of cellulose, xylan, and pectin. Most cellulosomal proteins were included in cluster B, C, or D in cellulose, xylan, and pectin, and the abundance of these proteins did not change significantly (Fig. 4a). On the other hand, in samples corresponding to glucose as a carbon source, about half of the cellulosomal proteins were included in cluster A. Almost all cellulosomal proteins were included in cluster E in case of galactomannan (Fig. 4a).

The cell-free cellulosomes released in galactomannan-cultured *C. cellulovorans* might play an important role in cell wall degradation. In the secreted proteins of galactomannan-cultured *C. cellulovorans*, the most increasing cluster included 19 cellulosomal proteins (Fig. 4b), and in the cellular proteins of galactomannan-cultured *C. cellulovorans*, almost all cellulosomal proteins showed a decreasing trend (Fig. 4a). In other *Clostridium* species, it is reported that cell-free cellulosomes exert a degradative activity [32, 33]. Cell-free cellulosomes are those which do not bind to cell surfaces [32]. However, cell-free cellulosomes have not been reported in *C. cellulovorans*. Cellulosomal proteins were shown to be more abundant than non-cellulosomal proteins in a previous secreted proteome analysis [16], and a higher xylanolytic activity was reported in the cell-free fraction than in the cell-attached fraction of *C. cellulovorans* [34]. Furthermore, in our analysis, the most increasing cluster of cellulose, galactomannan, and pectin included more cellulosomal proteins than other clusters (Fig. 4b). We postulate that the released cell-free cellulosomal proteins in *C. cellulovorans* degrade plant cell wall polysaccharides.

Among the total secreted proteins, 25 and 5 proteins in the cluster with the most increasing trend of cellulose were cellulosomal and non-cellulosomal proteins, respectively (Fig. 4b). We postulated that although cellulosomal proteins were mainly used, the non-cellulosomal proteins were also changed on a secondary level for cellulose degradation. *C. cellulovorans* was reported to significantly increase the non-cellulosomal protein content upon culture with phosphoric acid-swollen cellulose compared with cellobiose [16]. In contrast to cellulose, other polysaccharides-grown *C. cellulovorans* showed different proportions of cellulosomal and non-cellulosomal proteins, at the most increasing protein cluster among secreted proteins (Fig. 4b). When grown on glucose, two cellulosomal and two non-cellulosomal proteins were identified in the cluster with the most increasing trend in the secreted proteins. The coordination between cellulosomal proteins and non-cellulosomal proteins is important for the degradation of carbon sources [14, 16]. In samples corresponding to cellulose, clusters 1 and 2 contained 36 cellulosomal and 13 non-cellulosomal proteins, respectively. However, in samples corresponding to other polysaccharides, clusters 1 and 2 contained 24–26 cellulosomal and 17–26 non-cellulosomal proteins, respectively (Fig. 4b). Our data implies that only cellulosomal proteins play important roles in the degradation of cellulose. On the other hand, both cellulosomal and non-cellulosomal proteins might play an important role in the degradation of hemicellulose. In the clusters with the most increasing trends among the secreted proteins in xylan, pectin, and galactomannan, each carbohydrate degradation protein demonstrated degradation of every tested carbon source (Additional file 1).

In the *k*-means analysis of cellular proteins, proteins for the metabolism of cofactors and vitamins were ranked as top 1 in the cluster A of pectin and xylan. In a previous report, the abundance of proteins related to cobalamin metabolism in *C. cellulovorans* was found to increase when pectin was used as a carbon source [18]. Conforming to the previous report, our data also suggests that xylan and pectin increased similar cobalamin-related proteins (Additional file 1). The scope of our research could not reveal the role of cobalamin in xylan and pectin degradation or metabolism. The increase of cobalamin-related proteins might represent a secondary effect of degradation or metabolism of xylan and pectin. Further investigation into the underlying mechanisms needs to be performed in future research.

### Pathway analysis of cellular proteins

In the all carbon sources, the abundance of glycolysis related proteins in all polysaccharides did not change compared with glucose and TP1, but phosphofructokinase was increased about 2–4-folds in cellulose, galactomannan, and pectin among TP1, TP4, and TP5 (Additional file 4). Phosphofructokinase controls glycolysis, and its levels in *C. thermocellum* cultured using cellobiose, increased about 2-folds in the stationary phase compared with the exponential phase [35]. Our analysis confirmed this profile when *C. cellulovorans* was cultured on all carbon sources. In the pentose phosphate pathway, one transketolase (Clocel_1257) increased more than 2-folds at TP3, TP4, and TP5 compared with TP1 in pectin, and transaldolase (Clocel_1256) also increased approximately 2-folds in pectin. In contrast, the time course analysis of xylan indicated that one transaldolase (Clocel_1256) decreased 2-fold, but another transaldolase (Clocel_0591) increased 4-fold (Additional file 5). A previous research on *C. cellulovorans* showed that the abundance of proteins involved in xylan metabolism increased upon culture with xylan, whereas the abundance of proteins involved in pectin metabolism was observed when *C. cellulovorans* was cultured on galactomannan and pectin. [18]. The abundance levels of these proteins might reflect enzyme specificity or function for these two isozymes (transaldolase; Clocel_0591 and Clocel_1256). In the TCA cycle, the acetate-metabolizing pathway was activated in cultures grown on pectin compared with glucose. Pectin contains a few acetyl groups [7], and its acetyl group might be released due to the action of acetylesterase (Clocel_2252), which might explain the increase in levels of enzymes involved the acetate-metabolizing pathway (Additional file 6). Although different major plant cell wall polysaccharides were utilized as carbon sources, the central metabolic enzymes did not change significantly.

Next, we focused on the metabolism of major plant cell wall polysaccharides. A previous research on *C. cellulovorans* showed that the abundance of proteins involved in xylan metabolism increased upon culture with xylan, whereas the abundance of proteins involved in pectin metabolism was observed when *C. cellulovorans* was cultured on galactomannan and pectin. [18]. The abundance levels of these proteins might reflect enzyme specificity or function for these two isozymes (transaldolase; Clocel_0591 and Clocel_1256).

Galactokinase and galactose-1-phosphate uridylyltransferase, enzymes related with the galactomannan degradation pathway, were increased when *C. cellulovorans* was cultured using pectin (Additional file 8 b). Pectins usually contain rhamnogalacturonan-1, and we identified a rhamnogalacturonan-1-degrading enzyme in the secreted proteome analysis of galactomannan-cultured *C. cellulovorans* (Additional file 1). Based on this, we speculate that galactose derived from rhamnogalacturonan-1 is metabolized in this pathway.

Pectinesterase (Clocel_0211) and pectate lyase (Clocel_1172, Clocel_3834, and Clocel_1623) were not increased in the pool of cellular proteins compared with glucose when cultured on pectin (Additional file 9). However, pectinesterase and pectate lyase were identified as secreted proteins in pectin (Additional file 1). Pectin (polysaccharides of galacturonate) might be degraded to oligogalacturonides (oligosaccharides of galacturonate) by the secreted proteins.

### Co-abundance analysis of secreted proteins

In the co-abundance analysis, we found two medium size cluster, which included proteins with similar functions (Fig. 5). A previous report showed that the abundance of almost all proteins present in cluster (a) did not change, regardless of the carbon source [16]. Moreover, proteins in this cluster are predicted to have activity against cellulose [36], xylan [37], and galactomannan [38]. These proteins were identified corresponding to all tested carbon sources and have activity for multiple carbon sources. Thus, we speculate that these cellulosomal proteins might be the core enzymes in the degradation of cellulose and hemicellulose.

In *C. cellulolyticum*, the expression of cellulosomal protease inhibitors was shown to be important for cellulase activity [39]. In *C. cellulovorans*¸ cellulosomal proteinase inhibitors inhibit plant cysteine proteinases [40]. The proteinases identified in the current analysis were metalloproteases and a serine protease (Fig. 5). Plants secrete anti-bacterial peptides and proteins as a defense against pathogens [41, 42]. We suggest that *C. cellulovorans* secretes proteases and proteinase inhibitors in a coordinated way to protect its carbohydrate enzymes from plant or other bacterial proteases. Using this system, *C. cellulovorans* can degrade polysaccharides in plant cell walls without losing carbohydrate-degrading enzyme activity. We speculate that *C. cellulovorans* might degrade anti-bacterial peptides and proteins with secreted proteases, or proteases might act in protein turnover of secreted proteins. It is of course possible that the identified proteases exhibit other roles; however, we assume that here, the proteases share a similar role with protease inhibitors identified in co-abundance analysis.

## Conclusion

**Fig. 6:**
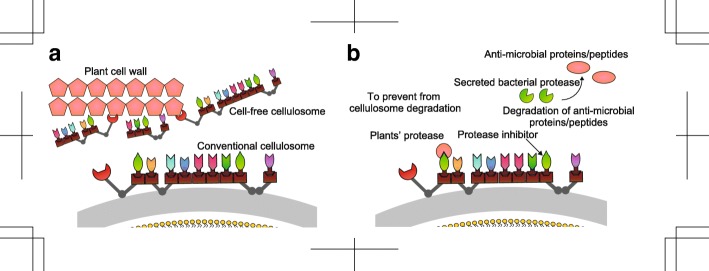
Proposal models obtained in this study. **a** Plant cell wall degrading model of cell-free cellulosome. Conventional cellulosome is anchored to the cell surface, but some studies reveal that cell-free cellulosome has an essential role in other cellulosome-producing *Clostridium* species. Cell-free cellulosome is a cellulosome which does not bind to the cell surface. Our analysis indicates that *C. cellulovorans* might secrete cellulosome to the supernatant or release cellulosome by partial degradation of cellulosome. We speculate that cell-free cellulosome might have a role as secreted proteins. **b** Co-abundance of proteases and protease inhibitors prevents cellulosome from degradation. Our analysis indicates that proteases and protease inhibitor are co-accumulated in all carbon sources. It was suggested that *C. cellulovorans*’ protease inhibitors inhibit plant proteases. Furthermore, our analysis indicates that some proteases are co-accumulated. We speculate that the secreted bacterial proteases might degrade anti-microbial proteins/peptides to protect *C. cellulovorans* from plant attack

We performed a temporal proteome analysis after culturing *C. cellulovorans* using five different carbon sources to investigate the temporal pattern of cellular and secreted proteome profiles. We identified a total of 1895 and 879 cellular and secreted proteins, respectively. The *k*-means clustering and co-abundance analyses suggested new evidence for polysaccharide degradation mechanisms and protease protection systems. We identified a few hitherto unidentified carbohydrate-degrading enzymes in the uniquely identified proteins from each carbon source. The *k*-means clustering in the cellular and secreted proteins suggested that cellulosomal proteins at the cell surface might be secreted or released by protease action (Fig. 6a). Co-abundance analysis implied that protease and protease inhibitors are accumulated simultaneously (Fig. 6b). These proteins might protect plant proteases and anti-microbial proteins/peptides. Our data are useful for further understanding of strategies and mechanisms applied by *C. cellulovorans* in the degradation and assimilation of major plant cell wall polysaccharides.

## Methods

### Bacterial strains and culture

*C. cellulovorans* 743B (ATCC35269) was purchased from the American type culture collection [9]. Culture medium was prepared as previously described [9], except for using the following five carbon sources: 0.3% glucose (Nacalai Tesque, Kyoto, Japan), 0.3% Sigmacell Cellulose Type 20 (Sigma, MO, USA), 0.3% xylan from Beechwood (SERVA Electrophoresis GmbH, Heidelberg, Germany), 0.3% locust bean gum from *Ceratonia siliqua* seeds (Sigma) used as galactomannan, and 0.3% pectin from apple, which is mainly composed from poly-d-galacturonate acid methyl ester (Sigma) without fractionation. *C. cellulovorans* was grown at 37 °C, pH 7.5, in 1-L glass anaerobic fermenters (three biological replicates). During the culture process, the culture media were fractionated and the ATP concentration was measured to determine growth curves [20]. For proteome analysis, 40 mL of culture media was collected at each predetermined TP and centrifuged at 6,000×*g* for 10 min at 4 °C, and the precipitated cells and supernatants were separated. The cell pellets were washed thrice with PBS (137 mM NaCl, 8.1 mM Na_2_HPO_4_, 2.68 mM KCl, 1.47 mM KH_2_PO_4_, pH 7.4; Nippon Gene, Tokyo, Japan). The washed cells and culture supernatants were flash-frozen and stored at - 80 °C until use.

### Preparation of cellular proteins for proteome analysis

Cellular proteins were obtained according to a previous protocol [43], with slight modifications. Briefly, the frozen cells were resuspended in 200 μL of lysis buffer (12 mM sodium deoxycholate; (Wako, Osaka, Japan) and 12 mM *N*-lauroylsarcosinate (Nacalai Tesque) in 50 mM Tris-HCl, pH 7.5) and disrupted by sonication at 250 W for 10 min (Bioruptor UCD-250 T; Cosmo Bio, Tokyo, Japan). The solution was centrifuged at 13,000×*g* for 20 min at 4 °C, and the supernatant was collected. Proteins were precipitated using methanol–chloroform precipitation, as described previously [44]. The precipitated sample was dissolved in 200 μL of reaction buffer (12 mM sodium deoxycholate, 12 mM *N*-lauroylsarcosinate, and 50 mM triethylammonium bicarbonate [TEAB; Sigma], pH 8.5) and reduced by adding 50 mM dithiothreitol (Nacalai Tesque, 1 M stock solution), and the reductive reaction was allowed to proceed for 30 min at 37 °C. Following incubation, 50 mM iodoacetamide (Wako, 500 mM stock solution) was added, and alkylation was allowed to proceed for 30 min at room temperature in the dark. Two micrograms of mass spectrometry grade lysyl endopeptidase (Wako) was added, and the solution was incubated at 37 °C for 4 h. Two micrograms of sequencing grade modified trypsin (Promega, Madison, WI, USA), and 1000 μL of TEAB were added to the solution, which was further incubated overnight at 37 °C. To remove the surfactant, 400 μL of ethyl acetate (Wako) was added to 400 μL of the digested solution. Eight microliters of trifluoroacetic acid (Wako) was added for acidification, and the mixture was shaken for 1 min, followed by centrifugation at 16,000×*g* for 2 min to separate aqueous and organic phases. The aqueous phase was collected and desalted using Monospin C18 (GL science, Osaka, Japan) according to the manufacturer’s protocol.

### Preparation of secretory proteins for proteome analysis

The supernatant was thawed on ice and subjected to ultrafiltration using an Amicon Ultra-15 centrifugal filter (Merck Millipore, MA, USA) to concentrate the secreted proteins as previously described [16, 17]. The concentrated proteins were precipitated using methanol–chloroform precipitation. Reductive alkylation, digestion of peptides, and their cleanup are described in the above section.

### Tandem mass tag labeling

The desalted samples were dissolved in 50 μL of TEAB. The dissolved peptide solution was labeled using a TMT 6-plex labeling kit (Thermo Fisher Scientific, MA, USA) according to the manufacturer’s protocol with a slight modification. The TMT-labeling reagents were dissolved in 41 μL of acetonitrile and mixed with 10 μL of TMT-labeling reagents to 100 μL. Each digested peptide solution was mixed with 10 μL of TMT-labeling reagents as described in Fig. 1. The tryptic digests of the lag, early log, middle log, late log, and stationary phases were labeled TMT-126, TMT-127, TMT-128, TMT-129, and TMT-130, respectively. In addition, an equal volume of all samples was mixed, and the mixture was labeled TMT-131 as an internal standard. After the labeling reaction, performed at room temperature for 60 min, the reactants were quenched by adding 8 μL of 5% hydroxylamine and lyophilized after mixing with 30 μL of each reactant. The dried samples were resolved in 30 μL of 0.1% formic acid.

### LC-MS/MS analysis

Proteome analysis was conducted according to a previous report [45], with slight modifications. Five microliters of the labeled samples were injected into the LC-MS/MS system. Proteome analysis was performed using an LC (Ultimate 3000; Thermo Fisher Scientific)-MS/MS (LTQ Orbitrap Velos Mass Spectrometer; Thermo Fisher Scientific) system equipped with a long monolithic silica capillary column [46] (490 cm long, 75 μm internal diameter) at a flow rate of 280 nL/min. A gradient was achieved by changing the ratio of two eluents: eluent A, 0.1% (v/v) formic acid, and eluent B, 80% acetonitrile containing 0.1% (v/v) formic acid. The gradient was started with 5% B, increased to 45% for 750 min, further increased to 95% B to wash the column for 140 min, returned to the initial condition, and was held for re-equilibration of the column. The separated analytes were detected using a mass spectrometer with a full scan range of 350–1500 *m/z* (resolution, 60,000), followed by 10 data-dependent higher-energy c-trap dissociation MS/MS scans acquired for TMT-reporter ions by using 35% normalized collision energy. The temperature of the ion transfer tube was set to 280 °C, and the dynamic exclusion was 180 s. An electrospray ionization voltage was set at 2.3 kV. Duplicate analyses were performed for each sample, and blank runs were inserted between different samples.

### Data analysis

Data analysis was performed using Proteome Discoverer 2.1 (Thermo Fisher Scientific). Protein identification was performed using MASCOT (Matrix Science, London, UK) against the *C. cellulovorans* protein database from NCBI, with a precursor mass tolerance of 20 ppm and a fragment ion mass tolerance of 50 mmu. Carbamidomethylation of cysteine and TMT 6-plex of N-term and lysine were set as fixed modifications, and oxidation of methionine was set as a dynamic modification. The data were filtered with the cutoff criteria of ≤0.01 (*q*-value), corresponding to a 1% false discovery rate on a spectrum level. Two criteria were applied for protein identification: (a) proteins with at least one unique peptide in all three biological replicates; (b) proteins with missing values only in the lag phase or with no missing values in the three biological replicates. Only those proteins which fulfilled both criteria were selected as identified proteins. The global median normalization was performed to normalize the amount of tryptic digests injected into the mass spectrometer in cellular protein. For *k*-means clustering of secreted proteins, all quantitative values were divided with log_10_ (Total ATP concentration of each TP) to normalize the amount of proteins in each TP. For the calculation of fold change relative to glucose, we used permutation-based false discovery rate calculation in Perseus [47], and the differences were plotted as fold changes. *k*-means clustering was conducted using R software. Functional annotation was performed using the KEGG BRITE annotation tool [24, 25]. The coefficient correlation of each quantitative value was calculated using Pearson’s correlation coefficient, and the protein co-abundance network was visualized using Cytoscape 3.5.0 [28].

## Additional files

Additional file 1: List of identified proteins under each condition. All proteins identified in our proteome analysis under each condition are shown. We show quantitative values and assigned *k*-means cluster, as represented in Fig. 4. CAZy family is obtained from CAZY Database (www.cazy.org). As an example, TP1_N1 indicates the sample obtained with time point 1 and biological replicates 1 (XLSX 1267 kb)

Additional file 2: Coefficients of correlation between different biological replicates. Coefficients of correlation were calculated between different biological replicates. As an example, GluTP1_N1 indicates protein samples prepared at time point 1 and biological replicate 1 (XLSX 12 kb)

Additional file 3: Detailed description of KEGG orthology analysis. We conducted KEGG orthology analysis to categorize the functional classes of each protein cluster with cellular proteins, and detailed information is shown. We highlighted the category of the functional class of each protein cluster described in the main manuscript. KEGG orthology term is shown in KEGG. At the # of identified proteins, the number of proteins categorized at the KEGG orthology term is described. At the percentage of total, we show the proportion of proteins belonging to each KEGG orthology term (XLSX 13 kb)

Additional file 4: Pathway analysis of glycolysis. Glycolysis pathway was constructed from KEGG. White box indicates the fold change of each protein relative to glucose at the same time point, and gray box indicates temporal profile of each protein relative to TP1 in each carbon sources. Each line indicates fold change or temporal profile (cellulose, red; xylan, purple; green, galactomannan; pectin, blue) (PDF 563 kb)

Additional file 5: Pathway analysis of pentose phosphate pathway . Pentose phosphate pathway was constructed from KEGG. White box indicates the fold change of each protein relative to glucose at the same time point, and gray box indicates temporal profile of each protein relative to TP1 in each carbon source. Each line indicates fold change or temporal profile (cellulose, red; xylan, purple; galactomannan, green; pectin, blue) (PDF 390 kb)

Additional file 6: Pathway analysis of tricarboxylic acid cycle. Tricarboxylic acid cycle was constructed from KEGG. White box indicates the fold change of each protein relative to glucose at the same time point, and gray box indicates temporal profile of each protein relative to TP1 in each carbon source. Each line indicates fold change or temporal profile (cellulose, red; xylan, purple; galactomannan, green; pectin, blue) (PDF 708 kb)

Additional file 7: Pathway analysis of xylan degradation and metabolism. Pathway of xylan degradation and metabolism was constructed from KEGG. White box indicates the fold change of each protein relative to glucose at the same time point, and gray box indicates temporal profile of each protein relative to TP1 in each carbon source. Each line indicates fold change or temporal profile (cellulose, red; xylan, purple; galactomannan, green; pectin, blue) (PDF 344 kb)

Additional file 8: Pathway analysis of galactomannan degradation and metabolism. (a) Pathway of mannan degradation and metabolism; (b) galactose degradation and metabolism pathway A. Each pathway was constructed from KEGG. White box indicates the fold change of each protein relative to glucose at the same time point, and gray box indicates temporal profile of each protein relative to TP1 in each carbon source. Each line indicates fold change or temporal profile (cellulose, red; xylan, purple; galactomannan, green; pectin, blue) (PDF 699 kb)

Additional file 9: Pathway analysis of pectin degradation and metabolism. Pathway of pectin degradation and metabolism was constructed from KEGG. White box indicates the fold change of each protein relative to glucose at the same time point, and gray box indicates temporal profile of each protein relative to TP1 in each carbon source. Each line indicates fold change or temporal profile (cellulose, red; xylan, purple; galactomannan, green; pectin, blue) (PDF 656 kb)

## Abbreviations

CAZy: Carbohydrate-active enzyme
CE: Carbohydrate esterase
GH: Glycoside hydrolase
GT: Glycosyltransferase
KEGG: Kyoto Encyclopedia of Genes and Genomes
PL: Polysaccharide lyase
TEAB: Triethylammonium bicarbonate
TMT: Tandem mass tag
TP: Time point
