# Supplementary figures and images for: Temporal proteome dynamics of Clostridium cellulovorans cultured with major plant cell wall polysaccharides
Source: BMC Microbiol. 2019 Jun 3;19:118. doi: 10.1186/s12866-019-1480-0 (PMC6547498; doi:10.1186/s12866-019-1480-0)

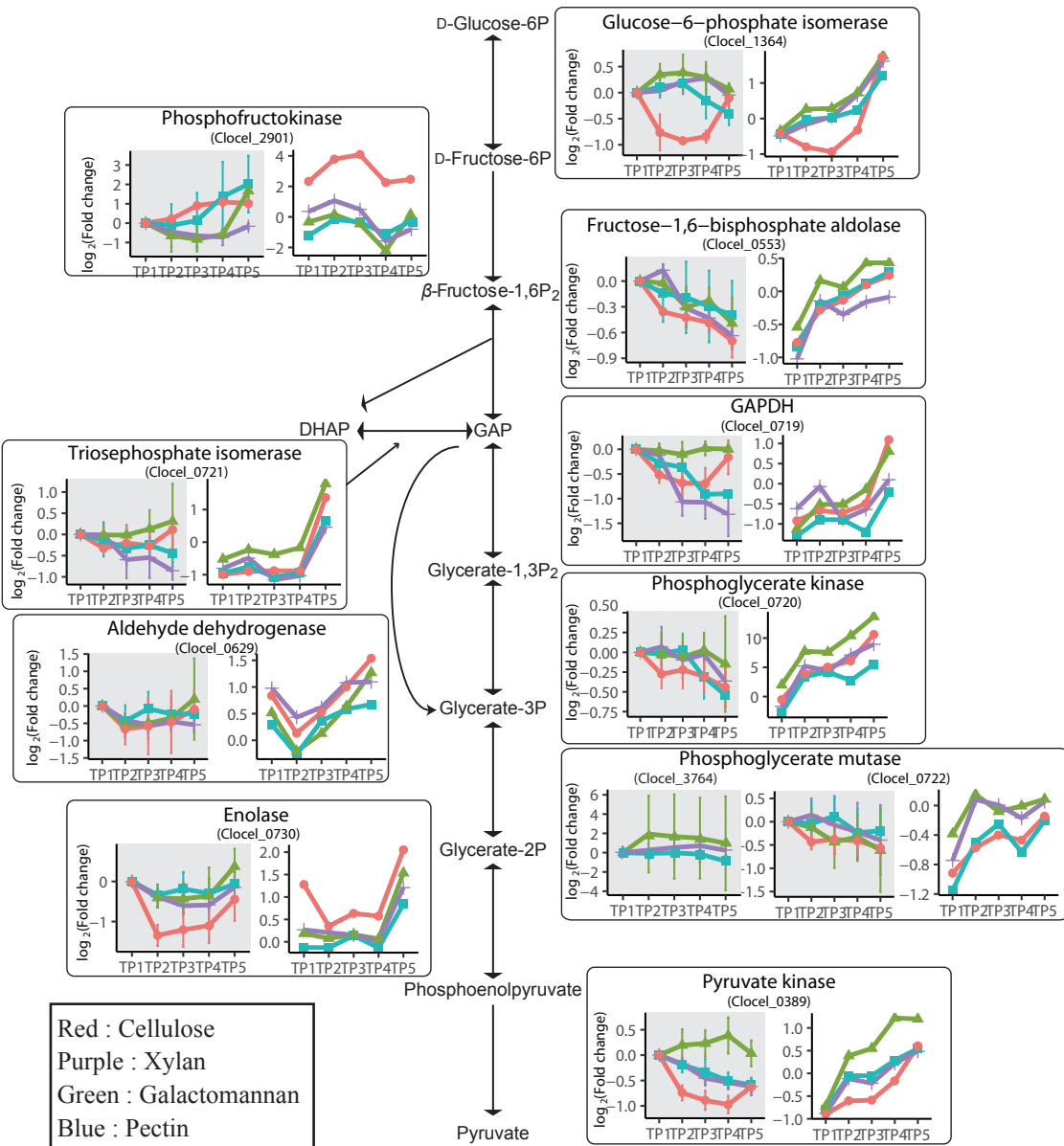

Supplement: Supplementary file 4 — Pathway analysis of glycolysis. Glycolysis pathway was constructed from KEGG. White box indicates the fold change of each protein relative to glucose at the same time point, and gray box indicates temporal profile of each protein relative to TP1 in each carbon sources. Each line indicates fold change or temporal profile (cellulose, red; xylan, purple; green, galactomannan; pectin, blue) (PDF 563 kb) [file 12866_2019_1480_MOESM4_ESM.pdf]

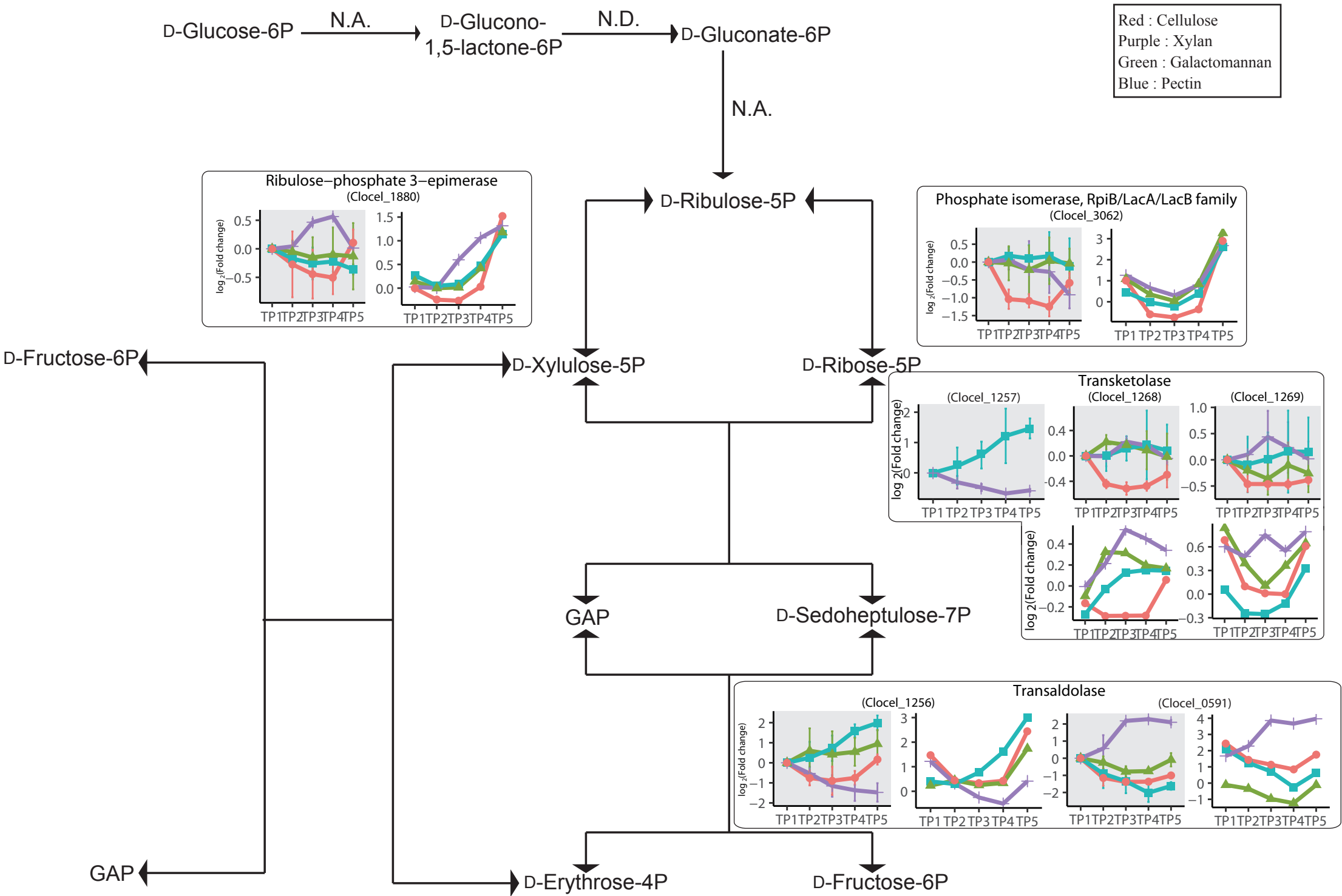

Supplement: Supplementary file 5 — Pathway analysis of pentose phosphate pathway . Pentose phosphate pathway was constructed from KEGG. White box indicates the fold change of each protein relative to glucose at the same time point, and gray box indicates temporal profile of each protein relative to TP1 in each carbon source. Each line indicates fold change or temporal profile (cellulose, red; xylan, purple; galactomannan, green; pectin, blue) (PDF 390 kb) [file 12866_2019_1480_MOESM5_ESM.pdf]

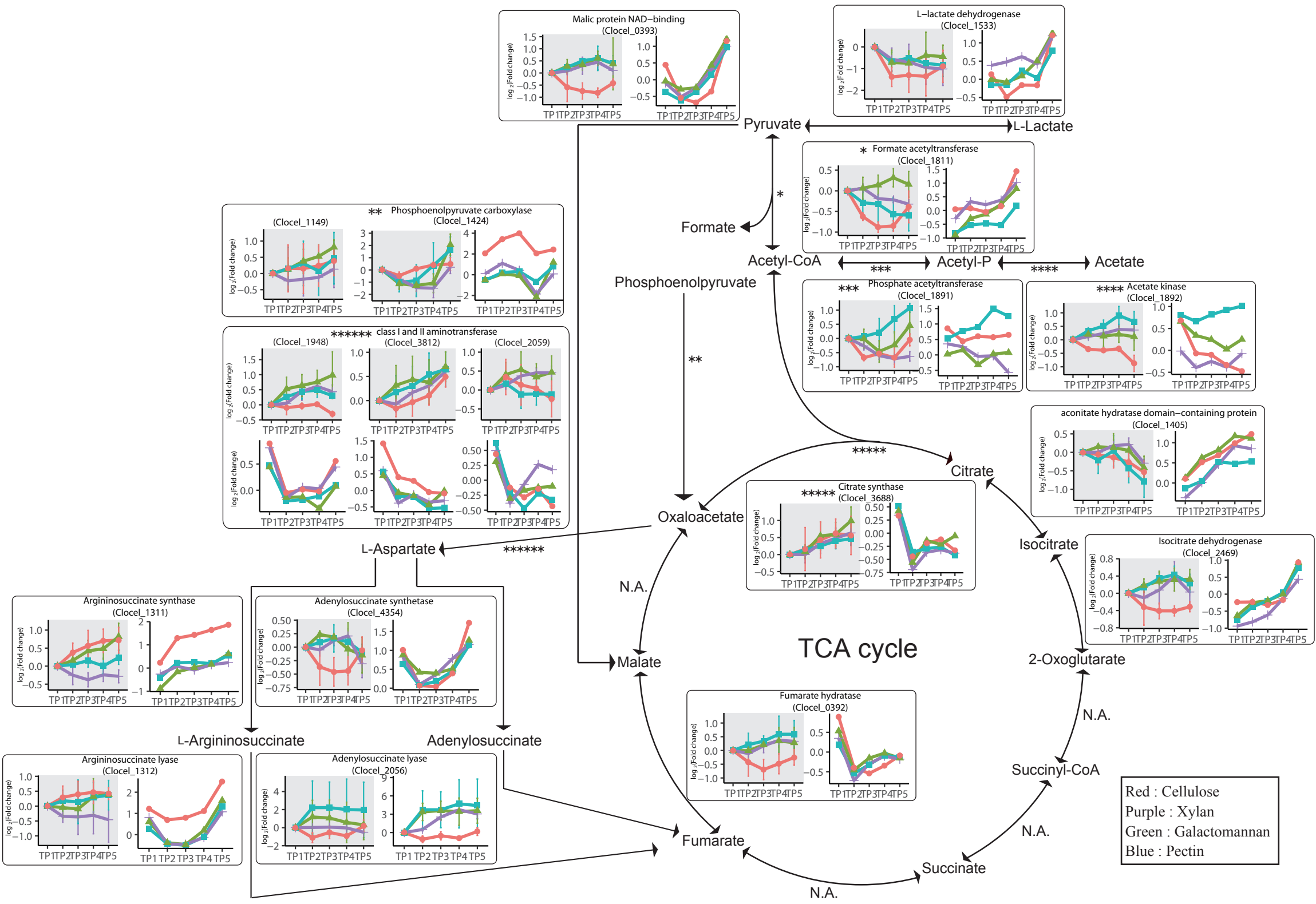

Supplement: Supplementary file 6 — Pathway analysis of tricarboxylic acid cycle. Tricarboxylic acid cycle was constructed from KEGG. White box indicates the fold change of each protein relative to glucose at the same time point, and gray box indicates temporal profile of each protein relative to TP1 in each carbon source. Each line indicates fold change or temporal profile (cellulose, red; xylan, purple; galactomannan, green; pectin, blue) (PDF 708 kb) [file 12866_2019_1480_MOESM6_ESM.pdf]

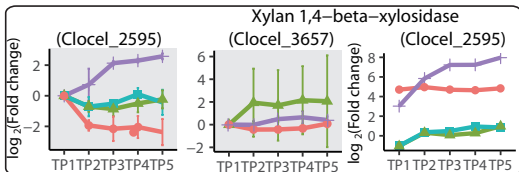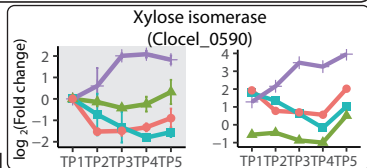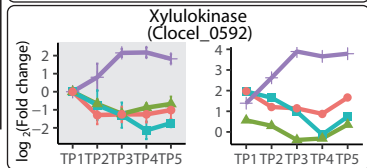

Red : Cellulose  
 Purple : Xylan  
 Green : Galactomannan  
 Blue : Pectin

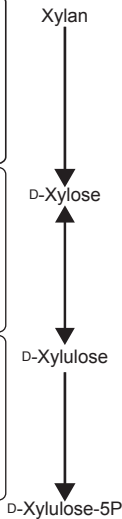

Supplement: Supplementary file 7 — Pathway analysis of xylan degradation and metabolism. Pathway of xylan degradation and metabolism was constructed from KEGG. White box indicates the fold change of each protein relative to glucose at the same time point, and gray box indicates temporal profile of each protein relative to TP1 in each carbon source. Each line indicates fold change or temporal profile (cellulose, red; xylan, purple; galactomannan, green; pectin, blue) (PDF 344 kb) [file 12866_2019_1480_MOESM7_ESM.pdf]

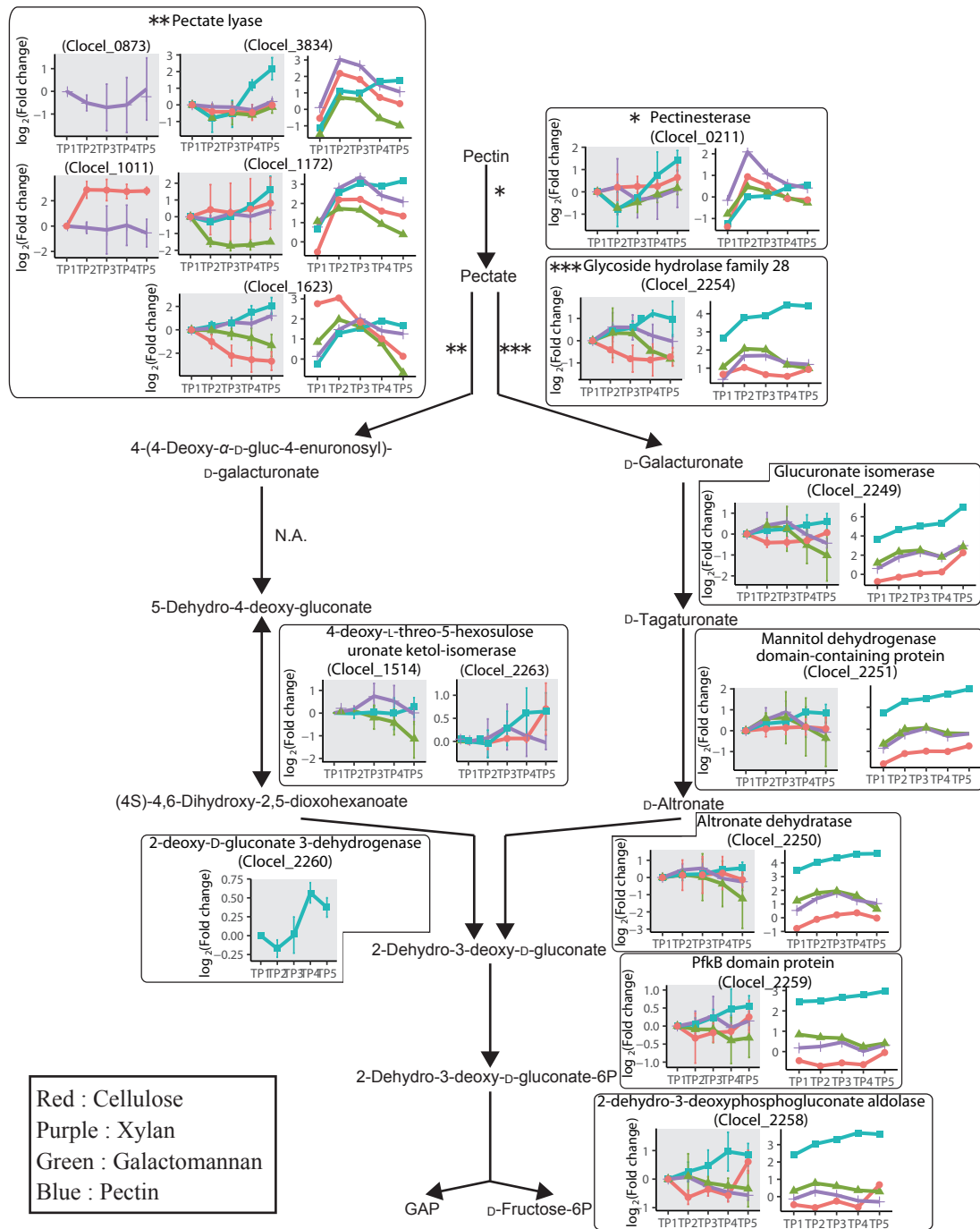

Supplement: Supplementary file 9 — Pathway analysis of pectin degradation and metabolism. Pathway of pectin degradation and metabolism was constructed from KEGG. White box indicates the fold change of each protein relative to glucose at the same time point, and gray box indicates temporal profile of each protein relative to TP1 in each carbon source. Each line indicates fold change or temporal profile (cellulose, red; xylan, purple; galactomannan, green; pectin, blue) (PDF 656 kb) [file 12866_2019_1480_MOESM9_ESM.pdf]
