# Supplementary material for: Temporal proteome dynamics of Clostridium cellulovorans cultured with major plant cell wall polysaccharides
Source: BMC Microbiol. 2019 Jun 3;19:118. doi: 10.1186/s12866-019-1480-0 (PMC6547498; doi:10.1186/s12866-019-1480-0)
Supplement: Supplementary file 8 — Pathway analysis of galactomannan degradation and metabolism. (a) Pathway of mannan degradation and metabolism; (b) galactose degradation and metabolism pathway A. Each pathway was constructed from KEGG. White box indicates the fold change of each protein relative to glucose at the same time point, and gray box indicates temporal profile of each protein relative to TP1 in each carbon source. Each line indicates fold change or temporal profile (cellulose, red; xylan, purple; galactomannan, green; pectin, blue) (PDF 699 kb) [file 12866_2019_1480_MOESM8_ESM.pdf]

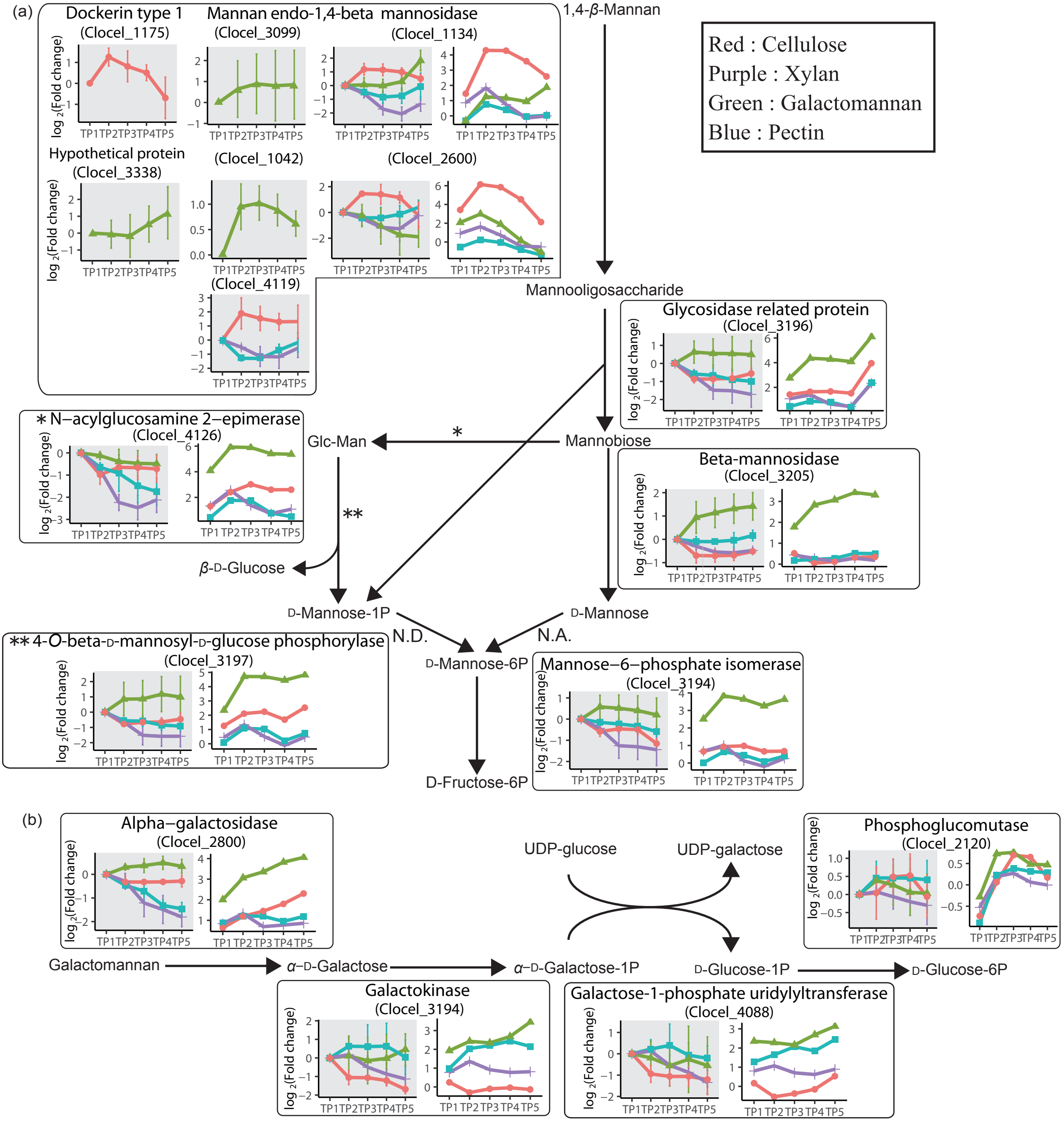

(a)
Red : Cellulose
Purple : Xylan
Green : Galactomannan
Blue : Pectin
Dockerin type 1
(Clocel_1175)
Mannan endo-1,4-beta mannosidase
(Clocel_3099)
(Clocel_1134)
Hypothetical protein
(Clocel_3338)
(Clocel_1042)
(Clocel_2600)
(Clocel_4119)
log 2(Fold change)
TP1 TP2 TP3 TP4 TP5
1,4-β-Mannan
Mannooligosaccharide
Glycosidase related protein
(Clocel_3196)
* N–acylglucosamine 2–epimerase
(Clocel_4126)
Glc-Man
Mannobiose
*
**
Beta-mannosidase
(Clocel_3205)
β-D-Glucose
D-Mannose-1P
D-Mannose
N.D.
N.A.
** 4-O-beta-D-mannosyl-D-glucose phosphorylase
(Clocel_3197)
D-Mannose-6P
Mannose–6–phosphate isomerase
(Clocel_3194)
D-Fructose-6P
(b)
Alpha–galactosidase
(Clocel_2800)
UDP-glucose
UDP-galactose
Phosphoglucomutase
(Clocel_2120)
Galactomannan
α–D-Galactose
α–D-Galactose-1P
D-Glucose-1P
D-Glucose-6P
Galactokinase
(Clocel_3194)
Galactose-1-phosphate uridylyltransferase
(Clocel_4088)
